# Ontogenetic allometry of the Beagle

**DOI:** 10.1186/1746-6148-9-203

**Published:** 2013-10-10

**Authors:** Daniela Helmsmüller, Patrick Wefstaedt, Ingo Nolte, Nadja Schilling

**Affiliations:** 1 Small Animal Clinic, University of Veterinary Medicine Hannover, Foundation, Bünteweg 9, 30559 Hannover, Germany; 2 Institute of Systematic Zoology and Evolutionary Biology, Friedrich-Schiller-University, Erbertstr. 1, 07743 Jena, Germany

**Keywords:** Scaling, Limb proportions, Body proportions, Bone growth, Serial homology, Body mass

## Abstract

**Background:**

Mammalian juveniles undergo dramatic changes in body conformation during development. As one of the most common companion animals, the time line and trajectory of a dog’s development and its body’s re-proportioning is of particular scientific interest. Several ontogenetic studies have investigated the skeletal development in dogs, but none has paid heed to the scapula as a critical part of the mammalian forelimb. Its functional integration into the forelimb changed the correspondence between fore- and hindlimb segments and previous ontogenetic studies observed more similar growth patterns for functionally than serially homologous elements. In this study, the ontogenetic development of six Beagle siblings was monitored between 9 and 51 weeks of age to investigate their skeletal allometry and compare this with data from other lines, breeds and species.

**Results:**

Body mass increased exponentially with time; log linear increase was observed up to the age of 15 weeks. Compared with body mass, withers and pelvic height as well as the lengths of the trunk, scapula, brachium and antebrachium, femur and crus exhibited positive allometry. Trunk circumference and pes showed negative allometry in all, pelvis and manus in most dogs. Thus, the typical mammalian intralimb re-proportioning with the proximal limb elements exhibiting positive allometry and the very distal ones showing negative allometry was observed. Relative lengths of the antebrachium, femur and crus increased, while those of the distal elements decreased.

**Conclusions:**

Beagles are fully-grown regarding body height but not body mass at about one year of age. Particular attention should be paid to feeding and physical exertion during the first 15 weeks when they grow more intensively. Compared with its siblings, a puppy’s size at 9 weeks is a good indicator for its final size. Among siblings, growth duration may vary substantially and appears not to be related to the adult size. Within breeds, a longer time to physically mature is hypothesized for larger-bodied breeding lines. Similar to other mammals, the Beagle displayed nearly optimal intralimb proportions throughout development. Neither the forelimbs nor the hindlimbs conformed with the previously observed proximo-distal order of the limb segment’s growth gradients. Potential factors responsible for variations in the ontogenetic allometry of mammals need further evaluation.

## Background

The physical development from a puppy to an adult dog is characterized by dramatic changes in body size and shape. Mammalian juveniles in general are not simply small copies of adults; they differ substantially in their body proportions and often appear clumsy in their movements (e.g., [1-3]). The juvenile body grows continuously while the musculoskeletal and nervous systems progressively mature. At the same time, juveniles have to perform in the same environment as adults, which results in unique challenges due to the differences in body size and conformation [4].

As the dog is one of the most common companion animals, the timeline and trajectories of its postnatal re-proportioning as well as the age at which it reaches adult proportions are of particular interest. Puppies are usually acquired by their new owners at the age of 9 to 11 weeks. For both the breeder and the potential buyer, the prospective physical development may be relevant when selecting a puppy. However, at the referral, the dogs are obviously not fully grown. Furthermore, during postnatal development, growth problems due to diet, injury or illness may occur and it is important to have reference values for the postnatal growth of the various body parts. A number of allometric studies are available for adult dogs; for example, comparing different breeds or examining historical or genetic transformations (e.g., [5-12]). Of the ontogenetic studies, some focused on pathological processes (e.g., [13,14]), while others documented either the physiological and pathological development of a single limb segment (e.g., [15-17]) or of several body parts [18-23]. Using x-ray in a longitudinal approach, Yonamine et al. [19] and Conzemius et al. [20] examined the growth of the forelimb or a part of it, respectively. Weise [18] followed the changes in body proportions among siblings in eight breeds and concluded that size differences among siblings are not due to differences in the duration of growth but growth rate. Schulze and colleagues [22,23] studied four breeds and a greater number of individuals per breed compared to Weise [18]; similarly, they observed that larger breeds differ from smaller breeds in their growth rates rather than growth duration. Salomon et al. [21] monitored 14 measurements of 37 Beagles during the first 13 months. They observed a higher growth rate in the hindlimbs than the forelimbs and no sex difference in growth termination. In contrast to the studies mentioned above [22,23] and in accordance with Hawthorne et al. [24], who investigated body mass development in different breeds, Salomon et al. [21] concluded that larger breeds grow for a longer time.

To investigate the ontogenetic scaling in dogs, this study monitored the allometry in Beagle siblings. The Beagle is a British breed and belongs to the hound group within the sporting breeds, which has been bred for pack hunting hares and rabbits. Nowadays, the Beagle is also a very popular family dog and a common laboratory animal. Within the breed, lines with different body sizes and proportions have been bred. Previous ontogenetic studies on Beagles worked with relatively small- to medium-sized lines (e.g., [19] adult body mass ca. 10 kg; [21] ca. 11 kg; [24] ca. 17 kg). In the current study, juveniles of a relatively large-bodied line were used (adult mass ca. 21 kg), allowing for a comparison of growth patterns among different-sized lines of the same breed.

During the evolution of mammals, fore- and hindlimbs underwent a profound reorganization that accompanied the transformation from a sprawled to a parasagittal limb posture. This resulted in a dissociation between serially and functionally homologous elements in the limbs (reviewed in [25]). The scapula was mobilized and is functionally analogous to the femur in mammals [26,27]. As a result, both fore- and hindlimbs can be described as three-segmented limbs arranged in a zig-zag-configuration with the most proximal elements (i.e., scapula, femur), the middle segments (i.e., brachium, crus) and the distal segments (antebrachium, pes) being functionally analogous due to their similar direction and amplitude of motion. Only a few allometric studies on adult (e.g., [25,28]) and juvenile mammals (e.g., [29-32]) paid heed to this evolutionarily 'new’ functional homology of the limb segments by taking the scapula into account. Comparing the results of these studies showed that in small mammals with a crouched limb posture the functionally homologous segments resemble each other more in their growth pattern than the serially homologous elements [32]. Specifically, three observations were made: First, the functionally homologous limb segments show more similar allometric coefficients than the serially homologous elements. Second, the limbs of various species such as rats, opossums, cuis or tree-shrews [32,33] show a proximo-distal gradient in their growth with the proximal segments growing the most and the distal segments growing the least (i.e., scapula and femur show higher allometric coefficients than antebrachium and pes). Third, the middle segment (i.e., brachium and crus) remains nearly constant in its proportion of the limb’s anatomical length to allow the animal to utilize self-stabilizing mechanisms [34]. Unfortunately, no ontogenetic study in dogs included the scapula in their measurements, hindering testing the proposed ontogenetic principles in dogs.

The aims of this study were 1) to test the observation that small and large dogs differ in rate but not duration of growth at the level of siblings, lines and breeds and 2) to examine the ontogenetic scaling of the Beagle in the light of the ontogenetic principles observed in other mammals.

## Methods

### Dogs

Six male Beagle siblings from the same litter (litter size: 7 males, 4 females) were used in this longitudinal study. The dogs were from a breeding colony of the University of Veterinary Medicine Hannover (Germany) and came to the Small Animal Clinic at the age of 9 weeks. One male and all females remained in the breeding colony and were not enrolled in this study to ensure similar husbandry conditions for the dogs investigated. All experiments were carried out in strict accordance with German Animal Welfare Regulations and were approved by the Ethics Committee of Lower Saxony, Germany.

Measuring started at 9 weeks and continued until the dogs were 51 weeks old. Data were collected weekly up to the age of 20 weeks, fortnightly up to 32 weeks and monthly until the end of the study. After that, only body mass was determined again at the age of 60 weeks. The dogs were kept and raised together in a group and under the same conditions, regarding, for example diet and exercise. Only one dog (#4) had to be regrouped at the age of 33 weeks, but its dietary plan and physical activity was comparable to that of its siblings. All dogs were vaccinated against distemper, hepatitis, canine parvovirus, leptospirosis and rabies at 9 and 12 weeks. However, between the age of 15 and 19 weeks, the dogs suffered from canine parvovirus and no measurements could be taken during this period. All puppies primarily experienced gastrointestinal upset and were treated immediately and aggressively in our clinics (i.e., fluid replacement, dietary restrictions, antiemetic and antibiotic therapy). As cell turnover in the gastrointestinal tract is rapid (1–3 days), intestinal malabsorption is short-lived and recovery from this enteric form is rapid [35].

At the age of about 40 weeks, all dogs were neutered. Between 32 and 51 weeks, occasionally smaller infections or injuries prevented the data collection from one or the other dog. During the study period, all dogs underwent two standard orthopedic investigations, one at 14 and one at 50 weeks of age, which confirmed that the dogs were healthy. The dogs were fed three times a day until the age of 44 weeks, afterwards twice a day. Portion size was about 1.9% of the dog’s body mass. At about 50 weeks, adult feed replaced the puppy feed. Over the course of the year when the dogs were investigated, their body index was in the normal range between 4 and 6 based on the body condition score (Nestlé Purina Pet Care Centre, St. Louis, MO, USA), in which values range from 1 to 9 (1–3 too thin; 4–5 ideal, 6–9 too heavy). For comparison, the parents were also measured when their offspring were about 32 weeks old. At this time, the sire was 7 years old and had a score of 7 and the dam was 6 years old and had a score of 6.

### Data collection and analyses

Body mass was determined to the first decimal using a traditional scale. A growth curve was constructed by plotting body mass against age using the Gompertz equation in the form: m_t_= m_max_exp(-exp^[-(t-c)/b]^), where m_t_ is mass at time t, m_max_ is mature body mass, b is proportional to duration of growth, c is the age at point of inflection (i.e., 36.8% of mature body mass) and t is age in weeks (for details, see [36]). Growth duration to reach 98% of the mature body mass was estimated as 4b+c. Similarly, 50% of growth duration was determined as 0.37b+c and 95% as 3b+c. All parameters were calculated for each dog and for the mean values for all dogs using a nonlinear regression program (NLREG; http://www.nlreg.com).

**Figure 1:**
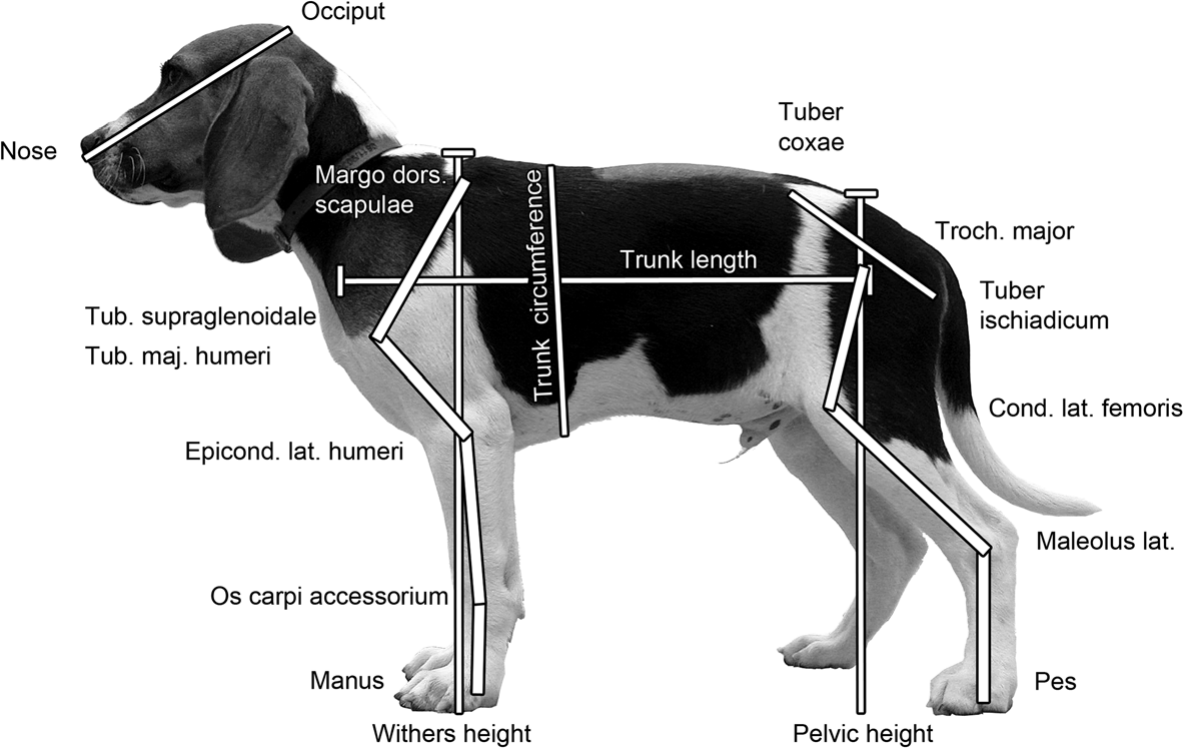
**Recorded measurements.** Photograph of dog #4 at the age of 15 weeks to illustrate the body and segment lengths measured. (The dog’s back was partially shaved for a joined study).

The lengths of the head, trunk and limb segments, trunk circumference as well as withers and pelvic heights were measured on the left body side using palpable skeletal landmarks and a traditional measuring tape (accuracy 5 mm, Figure 1). To reduce measurement errors, the measurements were always carried out by the same experienced experimenter (NS) and repeated three times per measurement. From these, means and the anatomical limb length (i.e., sum of the lengths of all segments) were calculated for further analysis. Correlation between the proportion of a respective segment of the anatomical limb length and age was calculated and tested for significance. To compare our results with previous findings [21], the Gompertz equation was also used to calculate the age when 95% of the final length of the brachium, antebrachium, femur and crus were reached.

Data analysis followed previous ontogenetic analyses [32,33]. For the allometric comparisons, the data were plotted on log-log scales (base 10) and regression lines were calculated by model II of the reduced major axis regression (RMA). Model II is to be preferred if variables, in this case body size parameters, could not be determined without error [37]. Besides, least-squares regression can lead to biased results if log-log bivariate regressions are used [38]. RMA regressions were calculated using Microsoft Excel (2000). The validity of the data obtained using Excel was previously tested and verified [32], and reevaluated for the current study using the software RMA (v. 1.17; http://www.bio.sdsu.edu/pub/andy/RMA.html). The exponent describing the slope of the regression curve is the allometric coefficient b. It indicates whether growth is isometric, negative or positive allometric. If a one-dimensional parameter (e.g., head length) is plotted *vs.* a three-dimensional one (e.g., body mass), isometry is given by b=0.333, negative allometry by b<0.333 and positive allometry by b>0.333. Comparing the same dimensions (e.g., two lengths), isometry is given by b=1.000, negative allometry by b<1.000 and positive by b>1.000. To test whether the allometric coefficients were significantly different from isometry, the 95% confidence intervals surrounding the slopes were calculated. If the interval overlapped with the slope, it was considered isometric. For comparisons among dogs, but also with previously published data from other mammals, so-called 'growth sequences’ were determined by sorting the slopes from the greatest to the lowest values. The slopes of two adjacent measurements were not considered different if their confidence intervals overlapped.

## Results

### Body mass

**Figure 2:**
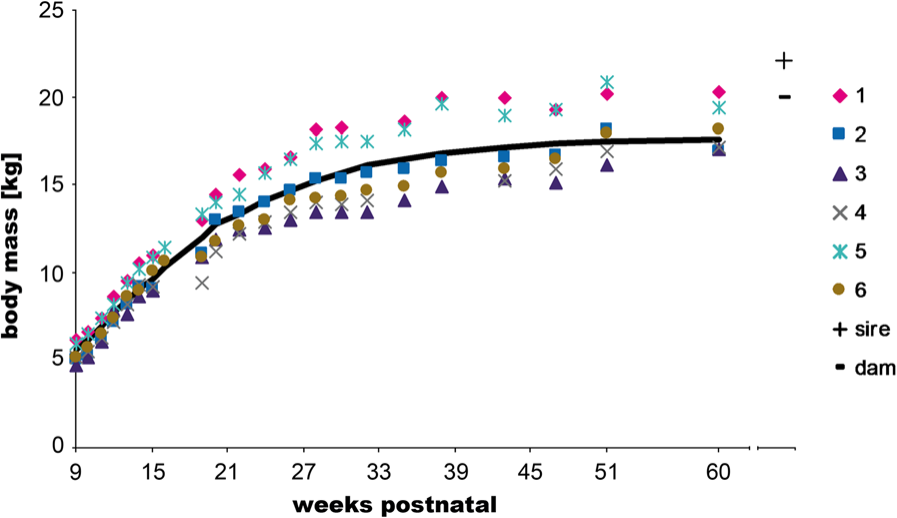
**Body mass development of the dogs studied and average growth curve estimated with the Gompertz function.** The parental data were added for comparison.

The dogs gained weight throughout the study period (Figure 2). The fit of the Gompertz equation to the body mass data was good (mean R^2^= 0.987). The estimated mean parameters were: Mature body mass m_max_=17.5±0.3 kg (individual dogs ranging between 16 kg and 20 kg), age at point of inflection c=11.1±0.3 weeks (ranging between 10.1 and 11.2 weeks) and the parameter proportional to growth duration b=9.18±0.7 (ranging between 8 and 10.6). On average, all dogs had reached 50% of their mature body mass with 14.5 weeks. Until the age of 15 weeks, log body mass increased linearly (R^2^=0.990). Mean age at 95% of the mature body mass was 39 weeks and at 98% 48 weeks. By the end of the study, no dog had reached the sire’s body mass (Figure 2), but as mentioned above, he was slightly overweight. Furthermore, dogs continue to gain muscle mass during their first years of life (see Discussion).

At week 9, dog #3 was the lightest individual (4.8 kg) and remained so until 51 weeks of age (16.1 kg). Similarly, the heaviest puppies at 9 weeks continued to be the heaviest dogs until week 51 (#1: 6.2 kg and 20.2 kg; #5: 6.0 kg and 20.9 kg). Interestingly, the relative body mass difference between the lightest and heaviest sibling (ca. 22% of body mass) persisted throughout the study. Between 15 and 19 weeks, some dogs showed only very little gain in body mass; however, they returned to their ontogenetic trajectory within a few weeks. Dog #4 did not gain any weight during this period, being the dog most affected by the parvovirus infection. He was back on his trajectory and among the sibling’s masses within 5 weeks after recovery.

### Body proportions

**Figure 3:**
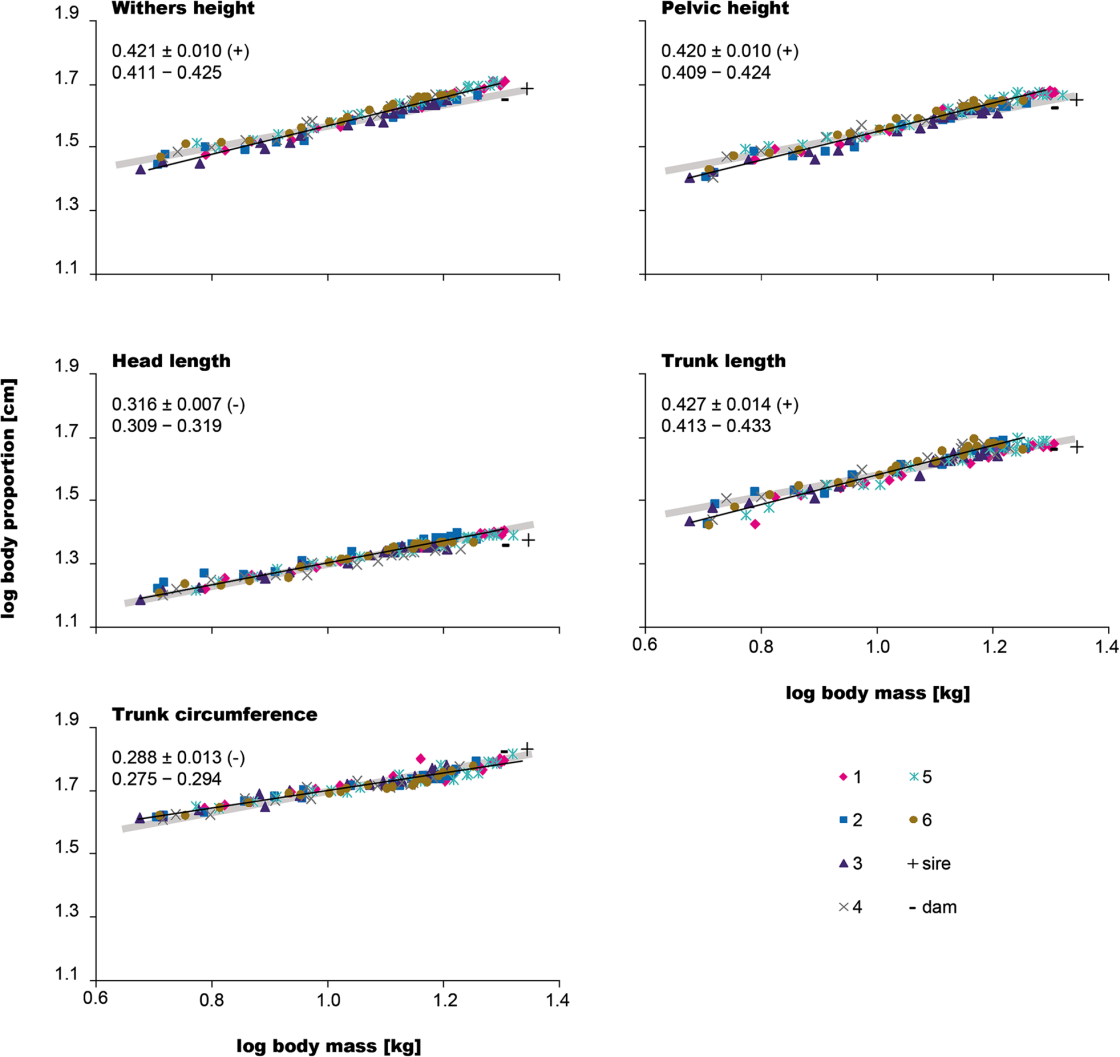
**Body proportions.** Logarithmic plots of withers and pelvic height, head and trunk lengths as well as trunk circumference *vs*. body mass for all dogs studied. The parental data were added in black for comparison. Black lines represent the regression lines fitted to the data of all juveniles; gray lines indicate isometry. Mean±SD of the allometric coefficients of the juveniles as well as the information of whether the respective parameter showed positive (+) or negative (-) allometry are given in the top left corner of each graph (first line). Numbers below indicate the UL and the LL of the 95% confidence intervals (second line). For allometric coefficients of each dog, see Table 1.

Compared to body mass, withers height, pelvic height, and trunk length exhibited positive allometry (Figure 3, Table 1). By the end of the study, all dogs had reached at least the mean withers and pelvic heights of the parents (46.5 cm and 43.5 cm, respectively). The only exception was dog #3, which remained smaller (44.3 cm and 40.7 cm) and also consistently showed the lowest values during the study. The sire’s withers and pelvic heights (48.3 cm and 45.0 cm) were surpassed by the two heaviest juveniles (#1: 51.3 cm and 47.7 cm; #5: 51.7 cm and 46.7 cm). Comparing the final heights with the values at 9 weeks shows that dog #5 grew the least of all dogs (36.8% and 33.2% increase in withers and pelvic height, respectively), whereas #1 grew the most as gauged by withers height (41.9%) but was in the middle range regarding its pelvic height increase (39.5%). Although dog #3 increased in his absolute withers height the least (17 cm), he was in the middle range regarding his relative increase (39.1%). Dog #4, despite suffering the most from the infection, gained the most in pelvic height of all dogs during the course of the study (41.6%). On average, 95% of the final height was reached at 212 days for withers height and 186 days for pelvic height.

**Table 1:** Individual parameters of body proportions for all siblings studied

		**hd**	**wi**	**ph**	**trl**	**trc**
Dog #1	Slope	0.340	0.446	0.434	0.424	0.287
	Intercept	0.963	1.119	1.117	1.135	1.417
	SD slope	0.009	0.013	0.012	0.020	0.021
	R	0.993	0.992	0.992	0.978	0.945
	95% UL	0.344	0.452	0.439	0.433	0.297
	95% LL	0.336	0.441	0.428	0.415	0.277
	Allometry	+	+	+	+	-
	Sequence	wi > ph = trl > hd > trc				
Dog #2	Slope	0.307	0.400	0.433	0.417	0.279
	Intercept	1.010	1.163	1.107	1.167	1.415
	SD slope	0.011	0.015	0.016	0.021	0.013
	R	0.987	0.985	0.986	0.975	0.978
	95% UL	0.312	0.406	0.440	0.426	0.284
	95% LL	0.302	0.392	0.425	0.408	0.273
	Allometry	-	+	+	+	-
	Sequence	ph = trl > wi > hd > trc				
Dog #3	Slope	0.331	0.434	0.438	0.412	0.296
	Intercept	0.970	1.123	1.102	1.164	1.405
	SD slope	0.013	0.016	0.018	0.018	0.016
	R	0.985	0.987	0.983	0.981	0.972
	95% UL	0.337	0.441	0.446	0.420	0.303
	95% LL	0.326	0.428	0.430	0.404	0.289
	Allometry	iso	+	+	+	-
	Sequence	ph = wi > trl > hd > trc				
Dog #4	Slope	0.278	0.417	0.427	0.436	0.293
	Intercept	1.015	1.165	1.133	1.154	1.400
	SD slope	0.013	0.015	0.022	0.026	0.020
	R	0.979	0.988	0.976	0.968	0.957
	95% UL	0.284	0.424	0.437	0.448	0.302
	95% LL	0.272	0.410	0.416	0.425	0.284
	Allometry	-	+	+	+	-
	Sequence	trl = ph = wi > trc = hd				
Dog #5	Slope	0.316	0.420	0.386	0.443	0.287
	Intercept	0.987	1.163	1.177	1.124	1.410
	SD slope	0.009	0.011	0.016	0.017	0.020
	R	0.992	0.993	0.982	0.984	0.950
	95% UL	0.320	0.425	0.393	0.450	0.295
	95% LL	0.312	0.415	0.379	0.436	0.278
	Allometry	-	+	+	+	-
	Sequence	trl > wi > ph > hd > trc				
Dog #6	Slope	0.335	0.400	0.425	0.468	0.270
	Intercept	0.966	1.183	1.135	1.117	1.420
	SD slope	0.014	0.013	0.013	0.022	0.013
	R	0.984	0.989	0.990	0.976	0.974
	95% UL	0.341	0.406	0.431	0.478	0.276
	95% LL	0.329	0.395	0.419	0.459	0.264
	Allometry	iso	+	+	+	-
	Sequence	trl >ph > wi > hd > trc				

Allometric coefficient (slope), intercept, standard deviation of the slope (SD slope), correlation coefficient (R) and the upper and the lower limit of the 95% confidence interval (95% UL, 95% LL) of all body proportions plotted against body mass on log-log scales (base 10). Isometry (iso) was given if the slope b=0.333 was within the confidence interval; otherwise, it was negative (-) or positive (+) allometry. For comparison, the growth sequence based on the slopes and the respective CIs is indicated for each dog in the last line.

*Abbreviations:* hd-head length, wi-withers height, ph-pelvic height, trl-trunk length, trc-trunk circumference.

The trunk length of the sire (47.0 cm) was reached or exceeded by all dogs except #3 (43.7 cm), who also did not reach the dam’s value (45.6 cm). Dog #5 had the longest trunk at 51 weeks (49.0 cm); he was also longer than #1 (47.8 cm), although #1 grew absolutely (21.2 cm) and relatively (44.0%) the most. The lightest puppy (#3) had the shortest trunk at 51 weeks (43.7 cm) and also grew the least during the study period (37.4%). Trunk circumference showed negative allometry relative to body mass for all dogs (Table 1). Mean trunk circumference of the parents was reached by none of the juveniles during the first 51 weeks (66.8 cm); dog #5 was the one who most closely approached that of the parents (65.3 cm).

Three dogs exhibited negative allometry regarding their head lengths relative to body mass, dog #3 and #6 showed isometry (b=0.331 and b=0.335), and dog #1 showed positive allometry (b=0.340; Figure 3). Dog #3 (22.3 cm) and #4 (22.2 cm) were the only ones at 51 weeks, which lagged behind when compared with the parents’ head lengths (mean 23.3 cm). Despite having a relatively short head, #3 showed the second greatest increase in head length during the study period. In accordance with his overall large body size, #1 was the one with the longest head (25.5 cm). Relative to trunk length, head length exhibited negative allometry for all dogs.

### Limb proportions

**Figure 4:**
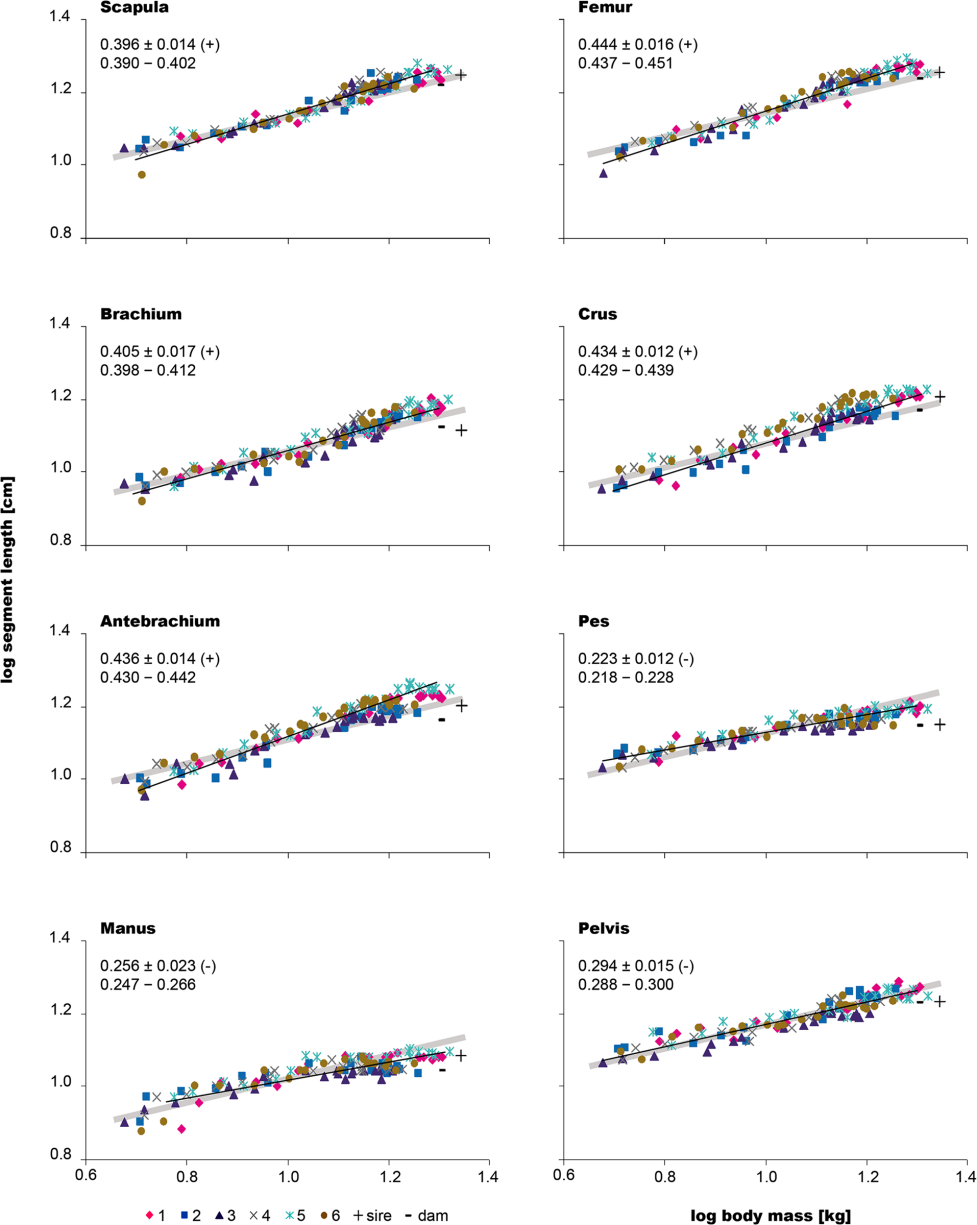
**Limb proportions.** Logarithmic plots of the segments of the fore- and hindlimb *vs*. body mass for all dogs studied. The parental data were added in black for comparison. Black lines represent the regression lines fitted to the data of all juveniles; gray lines indicate isometry. Mean±SD of the allometric coefficients of the juveniles as well as the information of whether the respective parameter showed positive (+) or negative (-) allometry are given in the top left corner of each graph (first line). Numbers below indicate the UL and the LL of the 95% confidence intervals (second line). For allometric coefficients of each dog, see Table 2.

Coefficients of segment lengths to body mass exhibited positive allometry for all dogs regarding scapula, brachium, antebrachium, femur and crus (Figure 4, Table 2). Pelvis, manus and pes showed negative allometry relative to body mass in all dogs, except the manus in dog #6 and pes in dog #4 (Table 2). Averaged across all individuals, the antebrachium had the highest allometric coefficient among the forelimb segments, followed by the brachium and the scapula (Figure 4). Thus, the growth sequence for the forelimb was ab>br=sc>ma (for individual sequences, see Table 2). In the hindlimb, femur and crus showed no significant difference, resulting in the growth sequence fe=cr>ps for all dogs.

**Table 2:** Individual parameters of limb segments for all siblings studied

		**Forelimb**				**Hindlimb**			
		**sc**	**br**	**ab**	**ma**	**pe**	**fe**	**cr**	**ps**
Dog #1	Slope	0.380	0.406	0.449	0.314	0.319	0.445	0.475	0.246
	Intercept	0.754	0.654	0.658	0.697	0.859	0.697	0.599	0.884
	SD slope	0.023	0.020	0.017	0.035	0.024	0.024	0.017	0.020
	R	0.963	0.977	0.986	0.872	0.945	0.972	0.988	0.938
	95% UL	0.390	0.415	0.457	0.329	0.330	0.456	0.483	0.255
	95% LL	0.369	0.397	0.442	0.298	0.308	0.435	0.467	0.237
	Allometry	+	+	+	-	-	+	+	-
	Sequence	ab > br > sc > ma				cr > fe > pe > ps			
Dog #2	Slope	0.386	0.392	0.436	0.227	0.322	0.437	0.438	0.220
	Intercept	0.756	0.665	0.663	0.789	0.859	0.707	0.633	0.907
	SD slope	0.023	0.024	0.023	0.028	0.024	0.022	0.022	0.013
	R	0.964	0.962	0.971	0.832	0.944	0.974	0.974	0.966
	95% UL	0.396	0.403	0.446	0.240	0.332	0.447	0.448	0.225
	95% LL	0.376	0.382	0.426	0.215	0.311	0.428	0.428	0.214
	Allometry	+	+	+	-	-	+	+	-
	Sequence	ab > br = sc > ma				cr = fe > pe > ps			
Dog #3	Slope	0.395	0.386	0.430	0.248	0.303	0.489	0.420	0.211
	Intercept	0.751	0.665	0.674	0.758	0.850	0.660	0.662	0.905
	SD slope	0.019	0.032	0.027	0.021	0.016	0.021	0.023	0.012
	R	0.976	0.930	0.959	0.925	0.970	0.982	0.971	0.966
	95% UL	0.403	0.400	0.442	0.257	0.310	0.498	0.429	0.216
	95% LL	0.386	0.373	0.418	0.239	0.296	0.480	0.410	0.205
	Allometry	+	+	+	-	-	+	+	-
	Sequence	ab > sc = br > ma				fe > cr > pe > ps			
Dog #4	Slope	0.437	0.415	0.432	0.243	0.334	0.454	0.408	0.250
	Intercept	0.713	0.657	0.695	0.781	0.839	0.708	0.709	0.874
	SD slope	0.020	0.027	0.024	0.027	0.025	0.021	0.019	0.018
	R	0.981	0.960	0.972	0.883	0.948	0.981	0.981	0.955
	95% UL	0.446	0.427	0.443	0.255	0.346	0.463	0.416	0.258
	95% LL	0.428	0.402	0.421	0.230	0.322	0.444	0.399	0.242
	Allometry	+	+	+	-	iso	+	+	-
	Sequence	sc = ab = br > ma				fe > cr > pe > ps			
Dog #5	Slope	0.404	0.419	0.468	0.242	0.267	0.454	0.422	0.233
	Intercept	0.738	0.650	0.661	0.794	0.917	0.694	0.690	0.907
	SD slope	0.024	0.020	0.020	0.020	0.021	0.022	0.015	0.017
	R	0.961	0.977	0.981	0.028	0.934	0.976	0.986	0.944
	95% UL	0.414	0.428	0.477	0.250	0.276	0.463	0.428	0.239
	95% LL	0.393	0.411	0.460	0.230	0.258	0.445	0.415	0.225
	Allometry	+	+	+	-	-	+	+	-
	Sequence	ab > br = sc > ma				fe > cr > pe > ps			
Dog #6	Slope	0.439	0.442	0.443	0.337	0.294	0.450	0.451	0.239
	Intercept	0.695	0.617	0.689	0.677	0.878	0.707	0.665	0.887
	SD slope	0.025	0.031	0.021	0.036	0.021	0.023	0.019	0.026
	R	0.965	0.947	0.976	0.873	0.942	0.971	0.982	0.872
	95% UL	0.450	0.456	0.452	0.352	0.304	0.460	0.459	0.250
	95% LL	0.428	0.429	0.434	0.322	0.285	0.440	0.443	0.228
	Allometry	+	+	+	iso	-	+	+	-
	Sequence	ab = br = sc > ma				cr = fe > pe > ps			

Allometric coefficient (slope), intercept, standard deviation of the slope (SD slope), correlation coefficient (R) and the upper and the lower limit of the 95% confidence intervals (95% UL, 95% LL) of all limb proportions plotted against body mass on log-log scales (base 10). Isometry (iso) was given if the slope b=0.333 was within the confidence interval; otherwise, it was negative (-) or positive (+) allometry. The growth sequences based on the slopes and the respective CIs are indicated for each dog in the last line. For comparisons with previously published results, see Table 4.

*Abbreviations:* sc-scapula, br-brachium, ab-antebrachium, ma-manus; pe-pelvis, fe-femur, cr-crus, ps-pes.

**Figure 5:**
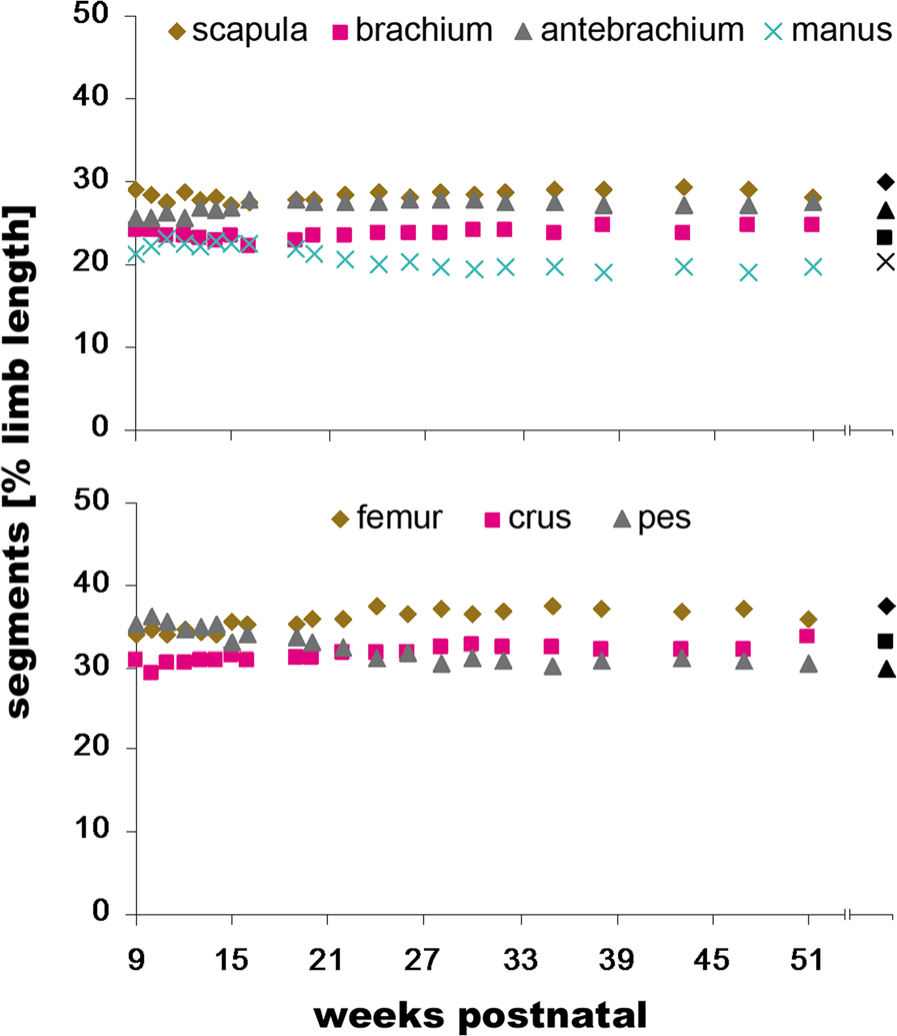
**Ontogenetic changes of relative segment lengths.** Relative segment lengths were determined as the proportion of the respective segment of the anatomical limb length (i.e., sum of scapula, brachium, antebrachium and hand as well as of femur, crus and pes, respectively). The parental data were added in black for comparison.

Proportions of the scapula and brachium of the anatomical forelimb length remained unchanged during development (sc: 29.0% *vs.* 28.1% and br: 24.0% *vs.* 24.8% at 9 and 51 weeks, respectively; Figure 5). In contrast, the antebrachium’s proportion was significantly correlated with age and increased from 25.8% at 9 to 27.5% at 51 weeks. In the hindlimb, the relative length of both femur and crus increased (fe: 33.8% *vs.* 35.9% and cr: 30.9% *vs.* 33.7% at 9 and 51 weeks, respectively). The distal elements, manus and pes, were inversely correlated with age (ma: 21.2% *vs*. 19.6% and ps: 35.2% *vs.* 30.4% at 9 and 51 weeks, respectively).

## Discussion

As only male siblings were investigated in this study, no implications for sex related differences can be drawn. However, previous studies found significant ontogenetic differences between sexes only for large breeds like the Great Dane or Bernese mountain dog but not for smaller breeds like the Beagle [19,21-23,39].

### Body mass

Comparing siblings of the same litters, Weise [18] observed wide ranges in the end dates of the growth of several skeletal parameters, indicating that the growth duration of siblings is not related with their final size. Albeit only a fraction of the siblings of one litter was studied herein, our findings support this observation. For example, the lightest dog did not reach its adult mass before the heavier ones and *vice versa*. Interestingly, the order among the siblings regarding body mass remained nearly unchanged during ontogeny. The lightest puppy at 9 weeks remained the lightest until the end of the study, and conversely, the heaviest puppies continued to be heavy throughout the study. This was true despite some puppies being affected by illness, because they quickly returned to their growth trajectory. Thus, our observation confirms Weise’s remark that a puppy’s size at 9 weeks is a good indication for its later size compared with its siblings.

Although the period of the maximal growth rate was not covered in the current study, because maximal weight gain occurs during the first 9 to 10 weeks in Beagles [21], log body mass still increased linearly up to the 15^th^ week in the Beagles studied herein. Likewise, Hawthorne and colleagues reported an exponential growth rate up to 14 to 16 weeks of age for the Beagle [24]. While our results are in agreement with the previous observation that 50% of the mature body mass is reached by the age of 14.8 weeks in a larger-bodied breeding line (17 kg, [24]), Salomon and colleagues, who studied a smaller-bodied line (11.8 kg), reported that their Beagles reached 50% of the mature body mass with only 7.1 weeks of age [21]. Compared with both previous studies, the time to reach mature body mass was estimated to be longer in the current study (95% of the mature mass at 35.1 weeks [21]*vs*. 38.6 weeks in this study; 99% after 41.9 weeks [24]*vs*. 98% after 47.8 weeks). However, a meaningful comparison among the studies is hindered because the mature body mass calculated for the dogs in this study probably underestimated their prospective adult body mass (i.e., calculated mass 17.5 kg *vs*. parents’ mean 21 kg). Dogs usually mature physically and gain muscle mass during their first years and thus after reaching their final body height. Although sample size in the current study was low and only a limited number of studies on different breeding lines is available, the comparison of the time lines of the body mass development of the different sized lines of the Beagle implies that body mass development varies among the breeding lines, particularly during the second half of development, and that larger-bodied lines tend to grow for a longer period. Substantial ontogenetic variation within breeds was also observed by Weise [18]. On the other hand, some variability in the growth patterns among breeds of the same body size category was reported by Hawthorne et al. [24].

In their comprehensive study, Hawthorne et al. [24] reported that 99% of the adult body mass was reached at about 10 months in toy, small and medium breeds (e.g., Papillon: 41 weeks, Cairn Terrier: 43 weeks, Beagle: 42 weeks) and between 11 to 15 months in large and giant breeds (e.g., Labrador Retriever & Great Dane: 52 weeks). In comparison, the Beagles in our study fall between the categories of medium and large breeds, given their 48 weeks to reach 98% of the mature body mass.

### Body proportions

Heads are relatively large in mammalian juveniles. Therefore, negative allometry was hypothesized in the current study and it is surprising that the head grew isometrically in two dogs and showed even positive allometry in one dog. Of the two heaviest dogs one showed negative allometry and the other showed positive allometry of the head’s length relative to body mass. The lightest dog’s head grew isometrically relative to its body mass, resulting in its head being relatively short at 9 weeks but within the normal range at 51 weeks. This is in contrast to Weise [18], who observed the shortest growth duration in the smallest siblings, resulting in smaller dogs having shorter heads. In addition to having relatively larger heads, puppies often appear plumper. As they approach adult size, the dogs become relatively longer and slimmer. For all dogs in this study, this is reflected by the negative allometry of the trunk circumference and the positive allometry of the trunk length compared with body mass and especially by the negative allometry of the head length *vs*. trunk length.

Due to the general maturation of the body in cranio-caudal direction (e.g., [40-43]), greater maturity of the forelimbs compared with the hindlimbs can be expected and was observed previously [21]. However, the allometric coefficients of the pelvic and withers height were similar in this study, which is probably related with its relatively late start at an age of 9 weeks, because higher growth rates were observed for the hindlimb during early development (e.g., between the 15^th^ and the 29^th^ day, [23]).

### Limb proportions

According to Salomon et al. [21], brachium and antebrachium of the Beagle reach 95% of their final length at 230 days and 217 days, respectively. In contrast, the brachium grew a bit longer in this study (mean: 254 days) and growth duration was shorter for the antebrachium (173 days). Femur and crus took less time to grow 95% of their final length in this study (mean: 180 and 206 days, respectively) compared with the earlier study (233 and 234 days [21]; end of growth according to [23]: 305 and 298 days). This clearly contradicts the observation from the body mass development, i.e., that larger-bodied lines grow for a longer period. Therefore, the Beagle line studied herein reached the final segment lengths relatively fast but gained weight (e.g., by increasing organ and muscles masses) for a longer period compared to other breeding lines.

Compared with other breeds, the Beagles in the current study also showed 95% of their final segment lengths earlier than Great Danes (ab: 238.9 days; fe: 262.5 days; cr: 272.9 days; [44]). The comparison of the growth among different breeds indicated that larger breeds grow at a higher rate but not necessarily for a longer period [22,23]. However, Weise [18] pointed out that the times until the dogs are fully grown may substantially differ among and within breeds as well as among and within litters. For example, she recorded times to full length from 140 to 243 days for the antebrachium and from 117 to 243 days for the crus in the poodle [18]. Similarly, variations of up to 52 days were observed among the siblings of the current study in reaching 95% of the final segment length. In summary, our results support Weise’s observations that larger siblings show higher growth rates and that the differences in the growth curves can be substantial among siblings.

### Comparison with other mammals

Based on the ontogenetic allometry of various species, it was observed that functionally homologous limb segments show more similar growth patterns than serially homologous segments in mammals [32]. The first finding in the former study was that the allometric coefficients were more similar between functionally homologous segments than serially homologous ones. In contrast to previous observations, the allometric coefficients of the functionally homologous segments were not comparable in dogs. Rather the growth of the antebrachium resembled that of the femur and the crus. Femur and crus showed higher allometric coefficients than scapula and brachium, respectively. This clearly contradicts the expectation of more similar allometric coefficients between functionally homologous limb segments. Nevertheless, the typical mammalian intralimb re-proportioning with the proximal elements showing positive allometry and the very distal ones exhibiting negative allometry was also observed in the Beagles studied herein (Table 3).

**Table 3:** Interspecific comparison of the ontogenetic allometry in various mammalian species

**Species**	**Forelimb**				**Hindlimb**				**Reference**
	**sc**	**br**	**ab**	**ma**	pe^**^	**fe**	**cr**	**ps**	
Domestic cat	+	+	+	-	+	+	+	-	[2]
Black-tailed jack rabbit	+	+	+	-	+	+	+	-	[30]
Western lowland gorilla	+	+			+	+			[45]
Mountain gorilla	+	+ ^m^ - ^f^			+	+ ^m^ - ^f^			[45]
European rabbit		+	+	iso		+	+	iso	[33]
Norway rat		+	iso	-		+	+	-	[33]
Grey short-tailed opossum		+	iso	-		+	+	iso	[33]
Long-tailed chinchilla		iso	-	-		+	+	-	[33]
Tree-shrew	+	+	-	-		+	iso	- iso	[32]
Cui	+	-	-	-		+	-	- -	[32]
Domestic dog (Beagle)	+	+	+	-	-	+	+	-	this study

(+) positive allometry, (-) negative allometry, (iso) isometry.

*Abbreviations:* sc-scapula, br-brachium, ab-antebrachium, ma-manus; pe-pelvis, fe-femur, cr-crus, ps-pes; m-male, f-female.

^**^Different measurements among studies (i.e., [30] pelvis, [2] ischium, [45] ilium). Note, two symbols in ma or ps indicate separate measurements for metacarpus or metatarsus and phalanges, respectively.

The second observation was that the proximal segments grow more than distal ones, i.e., the limb segments show a proximo-distal order in their growth gradients. While this is true for the fore- and hindlimbs of several mammalian species, in the Beagle it can neither be confirmed for the hindlimb nor for the forelimb (Table 4). Similar to the domestic cat [2], domestic pig [29], domestic rabbit [33], black-tailed jack rabbit [30], capuchin monkeys [46,47] as well as other dog breeds [22,39], the antebrachium grew more than the brachium in the Beagles studied herein. While the antebrachium also grew more than the scapula in this study, in both previous studies that included the scapula [29,30], the scapula grew more than any other segment (Table 4).

**Table 4:** Interspecific comparison of the growth sequences in various mammalian species

**Species**	**Forelimb**	**Hindlimb**	**Reference**
Grey short-tailed opossum	br > ab	fe > cr > ps	[33,48]
Long-tailed chinchilla	br > ab	fe > cr > ps	[33]
Norway rat	br > ab	fe > cr > ps	[33]
Tree-shrew	sc > br > ab	fe > cr > ps	[32]
Rhesus macaque	br > ab	fe > cr > ps	[49]
Human	br > ab	fe > cr	[50]
Brown-mantled tamarin	br > ab	fe > cr	[51]
African elephant	sc > br > ab	fe > cr	[31]
Asian elephant	sc > br > ab	fe > cr	[31]
Cui	sc > br > ab	fe > cr > ps	[32]
Western lowland gorilla	sc > br		[45]
Mountain gorilla	sc > br		[45]
Tuffed capuchin	ab > br	fe > cr > ps	[47]
White-fronted capuchin	ab > br	fe > cr > ps	[47]
Domestic pig	sc > ab > br	fe > cr	[29]
European rabbit	ab > br	fe = cr > ps	[33]
Domestic dog (Beagle)	ab > br = sc	fe = cr > ps	this study
Domestic dog (Great Dane)	ab > br	fe > cr	[39]
Domestic dog (Bernese Mountain dog)	ab > br	fe > cr	[22]
Domestic dog (Rottweiler)	ab > br	fe > cr	[22]
Geoffroy’s spider monkey	ab > br	cr > fe > ps	[46]
White-headed capuchin	ab > br	cr > fe > ps	[46]
Domestic cat	ab > br	cr > fe > ps	[2]
Black-tailed jack rabbit	sc > ab > br	cr > fe > ps	[30]
Virginia opossum	ab > br	ps > fe > cr	[48]

Growth sequences are based on the slopes observed in the respective studies.

The species in the upper half of the table show a proximo-distal growth sequence for both fore- and hindlimbs, while the species in the lower half show deviations from this sequence in either both or only the forelimb. Note that the separate measurements for metatarsus and phalanges were combined as pes herein.

*Abbreviations:* sc-scapula, br-brachium, ab-antebrachium, ma-manus; fe-femur, cr-crus, ps-pes.

The third observation concerned the proportions of the segments relative to limb length [32]. Simulations of three-segmented limb models showed that 1) proportions close to 1:1:1 are optimal for stability [34,52] and 2) mechanical self-stabilization of the model is achieved when the length of the middle segment remains constant, while the lengths of the proximal and distal segments were less critical to the model’s stability [34]. Accordingly, a greater variability in the proportions of the first and the third segment was observed across 189 mammalian taxa, while the middle element was less involved in alterations of the intralimb proportions [25]. In the current study, the Beagles showed forelimb proportions of 1.2:1.0:1.1 at 9 weeks and 1.1:1.0:1.1 as adults. Consistent with the model’s prediction, the brachium remained constant in its proportion of the limb’s anatomical length. In the hindlimb, the segment proportions were 1.1:1.0:1.1 at 9 weeks and 1.1:1.0:0.9 as adults. In contrast with the model, the crus increased in its relative length. However, overall, the intralimb proportions were near the optimum [53] in the juvenile and adult Beagles in this study and comparable to the segment ratios observed in other breeds of similar body size [54].

In summary, while some principles proposed in a previous study [32] held true for the Beagles studied herein, others did not. One reason may be that we compared growth patterns across all mammals for which data were available independent of their phylogeny, body size, limb posture, habitat or locomotor specialization. Given that these factors influence the intralimb proportions in mammals [25], they also probably influence growth patterns. Unfortunately, insufficient data are available at the moment to be able to assess the impact of these factors on the ontogenetic allometry of mammals. Furthermore, more studies assembling complete data sets for all limb segments are necessary to increase our understanding of the growth patterns in mammals in general and the dog in particular.

## Conclusions

At the age of one year, a Beagle has reached fully grown body height but not body mass. Up to about 15 weeks of age, Beagles grow particularly intensively, which should be considered regarding feeding and physical exertion. Compared with its siblings, a puppy’s size at 9 weeks is a good indication for its adult body size. Among siblings, growth duration may vary tremendously and seems not to be related to final body size. Within breeds, we hypothesize a longer duration to physically fully mature for larger-bodied lines. Throughout ontogeny, the Beagle displayed nearly optimum intralimb proportions. Neither the forelimbs nor the hindlimbs conformed with the proximo-distal growth sequence observed previously. Potential factors influencing the ontogenetic allometry of mammals such as phylogeny, locomotor behavior or body shape need to be evaluated in future studies.

## Abbreviations

ab: antebrachium; br: brachium; CI: Confidence interval; Cond: Condylus; cr: crus; dors: dorsalis; Epicond: Epicondylus; fe: femur; hd: head; iso: isometry; lat.: lateralis; LL: Lower limit of the CI; ma: manus; maj: majus; pe: pelvic length; ph: pelvic height; ps: pes; sc: scapula; SD: Standard deviation; trc: trunk circumference; trl: trunk length; Troch: Trochanter; Tub: Tuberculum; UL: Upper limit of the CI; wi: withers height.

## Competing interests

The authors declare that they have no competing interests.

## Authors’ contributions

DH, PW, IN and NS designed the study and approved the manuscript. DH and NS collected and analyzed the data and prepared the manuscript.
